# The effect of nutrition education sessions on energy availability, body composition, eating attitude and sports nutrition knowledge in young female endurance athletes

**DOI:** 10.3389/fpubh.2024.1289448

**Published:** 2024-03-14

**Authors:** Cansu Tektunalı Akman, Canan Gönen Aydın, Gülgün Ersoy

**Affiliations:** ^1^Department of Nutrition and Dietetics, Institute of Health Sciences, Medipol University, Istanbul, Türkiye; ^2^Department of Sports Medicine, University of Health Sciences, Baltalimanı Bone Diseases Training and Research Hospital, Istanbul, Türkiye

**Keywords:** nutrition education, sports nutrition knowledge, low energy availability, young female athletes, body composition, eating attitude

## Abstract

Nutrition knowledge plays a pivotal role in shaping dietary habits and food choices, particularly in the realm of sports nutrition. This study investigates the effects of a series of nutrition education sessions conducted by a registered dietitian on energy availability, various anthropometric measurements, eating attitudes, and sports nutrition knowledge in young female endurance athletes aged 15–18 years (football, basketball, volleyball) who engage in training for more than 10 h per week (*n* = 83). Participants were randomly divided into two groups with 45 individuals receiving six physical nutrition education lectures, and the remaining 38 participants receiving no nutrition education. Participants completed the low energy availability in females questionnaire (LEAF-Q), Eating Attitude Test (EAT-26), and Sports Nutrition Knowledge Questionnaire (SNKQ). Energy and nutrient intakes were evaluated through 3-day food records, while exercise energy expenditure was assessed using 3-day activity logs. All of the questionnaires were repeated after 6 months. At baseline, the prevalence of LEA among athletes was determined to be 63.8%. In the intervention group, energy availability (EA) and SNKQ scores increased, and LEAF-Q scores decreased significantly (*p* < 0.05). However, there was no significant change in EAT-26 scores between the two groups. Energy intake, weight, fat-free mass, and resting metabolic rate have been increased significantly in the intervention group (*p* < 0,05). These findings suggest that nutrition education proves beneficial in enhancing dietary intake, positively influencing body composition, and improving nutrition knowledge, ultimately contributing to increased energy availability in female athletes over the short term.

## 1 Introduction

For young athletes, the role of healthy and balanced nutrition is particularly important because of its impact on growth and development (1). Several studies have shown that young female athletes often fail to adhere to the recommended dietary guidelines for their sport and activity level (2), posing a risk for low energy availability (LEA) (2). Energy availability (EA) is defined as the amount of energy remaining and accessible for proper organism functions after subtracting the calories used for exercise from the diet, as described by the American College of Sports Medicine (3).

Clinical concerns associated with long-existing low EA include menstrual/libido, gastrointestinal and cardiovascular dysfunction, and compromised bone health, all of which can contribute to decreased sporting performance (4).

The prevalence of LEA among collegiate and young female athletes is reported to vary between 20 and 60%, depending on the type of sport and the level of competition (5–11). Several contributing factors, such as body image, the level of nutrition knowledge, and participation in weight-class sports can play a role in the presence of these observed energy deficiencies (12).

Nutrition education is a useful method in helping athletes to consume an adequate diet (7). The consumption of more fruit, vegetables, and carbohydrate-rich foods is more frequent in athletes with better nutrition knowledge than those without good nutritional understanding (8). This suggests that sports nutrition knowledge may increase awareness of appropriate dietary intake.

The purpose of sports nutrition is to help athletes acquire the appropriate food, energy, nutrients, and fluids to optimize athletic performance. Various obstacles including the stress and time commitments of being a full-time student and college athlete simultaneously, limited cooking skills, financial constraints, and inadequate nutrition knowledge impede proper fueling and recovery from sports. Nutrition interventions should be tailored individually considering the athlete’s specific event, personal goals, food preferences, and responses to various strategies (3).

Earlier interventions aimed at improving athletes’ nutrition knowledge have exhibited significant variations in their duration, content and whether they incorporated control groups (1) Until now, no previous studies have comprehensively assessed the effect of nutrition education on dietary intake, nutritional knowledge, risk of disordered eating and risk of low energy availability combined together in young female athletes. Additionally, there is a scarcity of data assessing nutritional adequacy in female athletes, especially those engaged in team sports. This study aims to address these gaps by evaluating the impact of a nutrition education intervention led by a registered dietitian on energy availability, anthropometric measurements, sports nutrition knowledge, and eating attitudes in adolescent female team sport athletes. The potential enhancements in nutrition knowledge, energy intake, eating attitudes, and body composition resulting from nutrition education will provide valuable insights to augment the existing literature.

## 2 Methods

This study is a randomized clinical trial (NCT06116097) with a control group and was conducted in Istanbul, Turkey. The data collection occurred from August 2019 to February 2020.

The G*Power 3.1.9.4 package program was used to calculate the sample size of the study. The sample size was calculated using the Mixed Measures Two-Way ANOVA analysis based on the difference between pre- and post-test sports nutrition knowledge measures in a related study. To calculate the sample size, the effect size was needed. In a related study conducted by Nascimento et al. (13), the impact of nutrition intervention on athletes’ nutrition knowledge was examined. In adolescent individuals, the pre-intervention sports nutrition knowledge was found to be 83.3 ± 18.70, and post-intervention was 92.2 ± 17.00. The effect size from this study was found to be 0.48. With an effect size of 0.48 (Cohen’s *f* = 0.48), a significance level of 0.05, a repeated measures correlation of 0.50, and the study’s power assumed to be 97%, it was determined that a total of 79 female athletes would be needed for the study.

In the initial phase, a total of 100 adolescent female athletes were included in the study and assigned to two groups of 50 each. However, 17 athletes were subsequently excluded: 4 athletes left their sports club, 3 sustained injuries and 10 athletes did not complete the entire procedure. Consequently, a total of 83 adolescent female elite athletes from three different sports clubs (football *n* = 34, basketball *n* = 16, and volleyball *n* = 33) aged 15 and 18 (mean 17.2 ± 2.0) were included in the study.

These participants were randomly assigned to either the intervention or control group through simple random sampling, ensuring statistical similarity in demographic characteristics. During the grouping process, sports clubs were selected from different regions of Istanbul, and care was taken to prevent any contact between athletes participating in the study. Forty-five participants underwent six physical nutrition education lectures, while the other group (*n* = 33) did not receive any nutrition education. All participants completed the Low Energy Availability in Athletes questionnaire (LEAF-Q), Eating Attitude Test (EAT-26), and Sports Nutrition Knowledge Questionnaire (SNKQ). Energy and nutrient intakes were evaluated based on 3-day food records and 3-day activity logs were analyzed to measure exercise energy expenditure.

**Figure 1:**
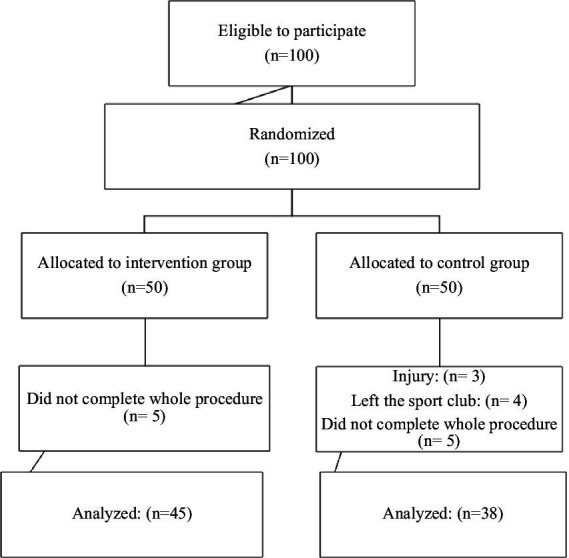
Trial CONSORT flow diagram.

Data assessments were conducted at two different time points (baseline and after 6 months). The participants provided written informed consent before participating in the study and had the option to withdraw at any time. The Medipol University ethical board approved the study (E.8234/196), ensuring adherence to the Declaration of Helsinki (see Figure 1).

### 2.1 Nutrition education

Nutrition education comprised six physical face-to-face 60-min sessions. These sessions were scheduled after coordinating with the coaches and were conducted every week by a registered dietitian in the sports clubs’ conference rooms. Each session covered a different subject including energy metabolism in sports, energy balance, nutrition before and after training, low energy availability, macro and micronutrients, hydration, and supplements. Participants were also provided with written information in the form of a printed booklet, allowing them to take notes during the sessions and review the material afterward.

### 2.2 Anthropometrics

Height was measured to the nearest centimeter using a portable stadiometer (Charder HM-200P, Taiwan). Participants were instructed to remove their shoes and socks for accurate height measurements. Body composition, including weight, body mass index (BMI), total body fat percentage, fat mass (FM), fat-free mass (FFM) was assessed using a multi-frequency electrical bioimpedance analyzer (BIA) (TANITA MC-780 P MA, Tanita Corp., Tokyo, Japan).

BIA is a method used for estimating body composition based on the principle of determining the electrical impedance of an electric current passing through the body (14). The electrical impedance (Z) comprises two components, resistance (R) and reactance (Xc). Reactance serves as a measure of Body Cell Mass (BCM), while resistance is a measure of total body water (14, 15). Using the determined impedance, various BIA parameters such as fat mass, fat-free mass, total body water, etc. can be estimated.

### 2.3 Energy availability

Energy availability (EA) was calculated using the formula (EI − EEE) / FFM, where EI is energy intake, EEE is exercise energy expenditure, and FFM is fat-free mass. EA values below 30 kcal/kg FFM were considered low EA between 30–45 kcal/kg was considered reduced and EA greater than 45 kcal/kg was considered optimal (16). Resting Metabolic Rate was calculated using the Cunningham equation: RMR = 500 + (22xFFM) (17). Fat-free mass (FFM) was derived from the BIA- measures.

### 2.4 Exercise energy expenditure

Exercise energy expenditure (EEE) was determined through 3-day activity logs both before and after the nutrition intervention during the active season. Participants documented their activities, including exercise duration and rest periods, for resistance and any non-club-based activities over 3 days. These activities were assigned Metabolic Equivalent (MET) values from the compendium of physical activities (18). Consistent with previous research (19, 20), to avoid overestimating energy expended during exercise, only activities with an intensity greater than 4.0 METs were considered in the calculation (21). Energy expenditure was calculated according to formula (22) Energy expenditure (calories/min) = 0.0175 x MET (from activity log) x weight (in kilograms) x time (in minutes). Subsequently, RMR that would have occurred regardless of exercise was subtracted from the thermic effect of exercise, ensuring that only the additional energy cost of exercise was included in the EEE (21).

### 2.5 Dietary intake

The mean daily intake of energy, macronutrients, and some micronutrients over the 3 days (including two weekdays and one weekend) was calculated. Participants utilized household measures to estimate their intake and the dietary data were entered into the BEBIS 6.1 nutrition analysis program (Beslenme Bilgi Sistemi, Turkey) (23). Daily energy, protein, carbohydrate, fat, and fiber intake were compared with the values recommended in the current American College of Sports Medicine (ACSM) Sports Nutrition Guidelines (3, 24).

### 2.6 Low energy availability questionnaire (LEAF-Q)

The 25-item LEAF-Q was utilized to assess the risk of LEA. The LEAF-Q has undergone validation in female athletes aged 18–39 who train at least five times per week, demonstrating acceptable sensitivity (78%) and specificity (90%) in classifying current energy availability (25). In line with the original validation study, participants completed a paper version of the LEAF-Q to ensure the maintenance of validity and reliability. Scoring followed the criteria established in the original validation study, categorizing individuals who scored ≤7 as ‘not at risk’ of LEA, and those scoring ≥8 as ‘at risk’ of LEA (25).

### 2.7 Eating attitude test (Eat-26)

EAT-26 was employed to assess the risk of eating disorders a tool utilized across various populations, including athletes (26, 27). Following the developers’ recommendations, scores of 20 or above on the EAT-26 assessment were indicative of eating disorder behaviors (28). Scores below 20 were considered low risk for eating disorder behaviors (28).

### 2.8 Sports nutrition knowledge questionnaire (SNKQ)

Participants completed the 88-item Sports Nutrition Knowledge Questionnaire (SNKQ) both before and after the intervention (28). The SNKQ has undergone assessment for validity (content and construct) and reliability (test–retest), demonstrating high construct validity and good test–retest concordance, thus confirming its suitability for determining sports nutrition knowledge. The SNKQ compromises five sub-sections (general nutrition concepts, fluid, recovery, weight control, and supplements). Participants were awarded one point for each correct answer, while an ‘unsure’ or incorrect response received zero points. Scores for each subsection and the total score were calculated based on the number of questions answered correctly with a maximum total score of 83.

### 2.9 Statistical analyses

All statistical analyses were conducted using the Statistical Package for Social Sciences (SPSS 28.0, Chicago, IL, United States). Results were reported as mean ± standard deviation (SD) for normally distributed variables and median and interquartile range (IQR) for non-normally distributed variables. Group comparisons of the baseline characteristics were conducted using the independent samples t-test for normally distributed data, and the Mann–Whitney test for non-normally distributed data. Data were analyzed by mixed model ANOVA followed by *post hoc* pairwise comparisons using the Bonferroni adjusted significance when group-by-time interaction was identified between groups (α = 0.05). The effect sizes were expressed as partial eta-squared values within mixed measures ANOVA squared (η^2^_p_; small ≥0.01, medium ≥0.06, large ≥0.14).

## 3 Results

A total of 83 girls aged between 15 and 18 years participated in the study. Table 1 shows the baseline characteristics of study participants, categorized by control and intervention groups.

**Table 1:** Baseline characteristics of study participants divided by control and intervention group.

	Control Group Mean. ± sd (*n* = 38)	Intervention Group Mean. ± sd (*n* = 45)	*p*
Age	17.2 ± 2.6	17.1 ± 1.5	0.528^m^
Height (cm)	169.5 ± 6.7	167.4 ± 9.5	0.229^t^
Weight (kg)	59.3 ± 8.9	58.6 ± 8.6	0.985^m^
Sport experience (year)	5.1 ± 2.2	6.3 ± 3.4	0.327^m^
EEE (kcal)	768.3 ± 197.2	633.8 ± 295.0	0.002^m^
TEE (kcal)	2,735,1 ± 359,8	2,896,5 ± 476,7	0.120^m^
BMI (kg/m^2^)	19.2 ± 5.8	19.4 ± 5.7	0.780^m^
BF (%)	19.2 ± 5.8	19.4 ± 5.7	0.780^m^
FM (kg)	11.7 ± 5.1	11.6 ± 4.6	0.871^m^
FFM (kg)	47.5 ± 5.0	47.0 ± 5.7	0.852^m^
RMR (kcal)	1545.9 ± 110.8	1533.1 ± 124.9	0.852^m^
EI (kcal)	2050,7 ± 498,9	1739,7 ± 396,6	0.0005^m^
EA (kcal/kg/FFM)	27,3 ± 12,2	24,1 ± 10,7	0,112^m^

^t^Independent sample *t* test / ^m^ Mann–whitney *u* test. BMI, Body Mass Index, BF, Body Fat Percentage, FM, Fat Mass, FFM, Fat-Free Mass, RMR, Resting Metabolic Rate, EI, Energy Intake, TEE, Total Energy Expenditure, EEE, Exercise Energy Expenditure, EA, Energy Availability. Values are expressed as mean ± standard deviation.

Changes in anthropometric parameters before and after the intervention are shown in Table 2.

**Table 2:** Changes in anthropometric parameters and energy expenditure measures before and after the intervention for both groups.

Parameter	Group	Pre	Post		*F*	Sig.	Partial Eta Squared
BMI (kg/m^2^)	Interv.	20,81 ± 2,16	21,23 ± 1,99	Time	5,84	0.02	0,07
	Control	20,47 ± 2,46	20,44 ± 2,55	Time*group	7,55	0.01	0,09
	Total	20,68 ± 2,28	20,91 ± 2,25	Group		0.26	
BF (%)	Interv.	19,12 ± 6,23	19,54 ± 5,59	Time	0,01	0.93	
	Control	18,98 ± 6,39	18,62 ± 6,07	Time*group	1,38	0.24	
	Total	19,06 ± 6,26	19,17 ± 5,77	Group		0.23	
FM (kg)	Interv.	11,64 ± 4,59	12,05 ± 4,65	Time	0,14	0.71	
	Control	11,74 ± 5,11	11,16 ± 5,22	Time*group	4,74	0.03	0,06
	Total	11,68 ± 4,77	11,70 ± 4,87	Group		0.70	
FFM (kg)	Interv.	46,96 ± 5,68	47,71 ± 5,36	Time	6,57	0.01	0,08
	Control	47,54 ± 5,04	47,79 ± 5,48	Time*group	1,64	0.20	
	Total	47,19 ± 5,41	47,74 ± 5,38	Group		0.78	
RMR (kcal)	Interv.	1,518,57 ± 132,94	1,531,04 ± 143,00	Time	3,45	0.07	
	Control	1,525,52 ± 118,49	1,539,08 ± 141,45	Time*group	0,01	0.94	
	Total	1,521,33 ± 126,70	1,534,24 ± 141,57	Group		0.800	
TEE (kcal)	Interv.	2,867,11 ± 467,44	2,892,84 ± 492,93	Time	3,91	0.06	
	Control	2,698,80 ± 364,91	2,723,67 ± 392,32	Time*group	0,00	0.97	
	Total	2,800,19 ± 435,20	2,825,58 ± 460,65	Group		0.09	
EEE (kcal)	Interv.	633,84 ± 295,01	645,48 ± 301,79	Time	1,88	0.17	
	Control	768,28 ± 197,24	763,26 ± 197,00	Time*group	11,88	0.00	0,13
	Total	687,30 ± 267,53	692,31 ± 270,06	Group	5,72	0.03	0,10
EA (kcal/kg/FFM*)*	Interv.	24,11 ± 10,69	32,24 ± 12,14	Time	13,14	0.00	0,14
	Control	27,25 ± 12,25	27,19 ± 12,89	Time*group	13,53	0.00	0,14
	Total	25,36 ± 11,37	30,23 ± 12,61	Group		0.69	

A significant group-by-time interaction effect was found for BMI (*F* (1,81) = 7.55, *p* < 0.05, partial eta squares = 0.09; medium). Following Bonferroni correction for pairwise comparisons, it was observed that BMI in the experimental group increased from pre-intervention (20.81 + −2.16) to post-intervention (21.23+/1.99) (*p* = 0.01). A significant group-by-time interaction effect was found for fat mass (*F* (1,81) = 4.74; *p* < 0.05, Partial Eta Squared = 0.06; medium). Following Bonferroni correction for pairwise comparisons, it was observed that fat mass in the experimental group increased from pre-intervention (11.64 ± 4.59) to post-intervention (12.05 ± 4.65) (*p* = 0.01).

A significant group-by-time interaction effect was found for EEE (*F* (1,81) = 11.88; *p* < 0.05, Partial Eta Squared = 0.13; medium). Following Bonferroni correction for pairwise comparisons, it was observed that EEE in the experimental group increased from pre-intervention (633.84 ± 295.01). to post-intervention (645.48 ± 301.79) (*p* = 0.01).

A significant group-by-time interaction effect was found for EA (*F* (1,81) = 13.53; *p* < 0.05, Partial Eta Squared = 0.14; large). Following Bonferroni correction for pairwise comparisons, it was observed that EA in the experimental group increased from pre-intervention (24.11 ± 10.69) to post-intervention (32.24 ± 12.14) (*p* = 0.01). Additionally, there was no group-by-time interaction for Body Fat Percentage, FFM, RMR, and TEE.

Changes in energy, macro, and micronutrient intakes of participants before and after the intervention are presented in Table 3.

**Table 3:** Energy, macronutrients, and micronutrient intakes of participants before and after the intervention.

Dietary intake	Group	Pre	Post		*F*	Sig.	Partial Eta Squared
EI	Interv.	1739,71 ± 396,61	2046,12 ± 447,98	Time	17,25	0,00	0,18
	Control	2050,74 ± 498,93	2034,82 ± 469,33	Time*group	20,18	0,00	0,20
	Total	1863,37 ± 463,24	2040,87 ± 457,04	Group		0,26	
Protein (g)	Interv.	70,66 ± 22,06	84,76 ± 22,53	Time	9,06	0,00	0,10
	Control	77,03 ± 21,64	78,09 ± 17,89	Time*group	6,71	0,01	0,08
	Total	73,19 ± 21,98	82,11 ± 20,95	Group		0,97	
Carbohydrate (g)	Interv.	184,36 ± 54,65	228,04 ± 57,09	Time	15,72	0,00	0,16
	Control	212,04 ± 77,35	221,57 ± 66,70	Time*group	6,48	0,01	0,07
	Total	195,50 ± 65,73	225,44 ± 60,82	Group		0,39	
Fat (g)	Interv.	76,52 ± 33,64	82,80 ± 25,91	Time	0,12	0,73	
	Control	88,58 ± 28,84	80,28 ± 17,49	Time*group	6,17	0,02	0,07
	Total	81,32 ± 32,19	81,80 ± 22,85	Group		0,38	
Fiber (g)	Interv.	30,10 ± 16,14	27,83 ± 9,90	Time	2,23	0,14	
	Control	29,20 ± 16,26	26,06 ± 9,43	Time*group	0,06	0,81	
	Total	29,74 ± 16,09	27,13 ± 9,70	Group		0,57	
Calcium (mg)	Interv.	1,153,50 ± 511,45	1,096,04 ± 431,65	Time	3,45	0,07	
	Control	1,164,03 ± 706,06	1,009,33 ± 392,14	Time*group	0,72	0,40	
	Total	1,157,69 ± 592,35	1,061,57 ± 416,14	Group		0,70	
Iron (mg)	Interv.	14,87 ± 7,05	13,32 ± 5,43	Time	5,01	0,03	0,06
	Control	16,76 ± 9,47	14,36 ± 3,34	Time*group	0,23	0,63	
	Total	15,62 ± 8,10	13,73 ± 4,71	Group		0,22	
Water (mL)	Interv.	2,130,90 ± 904,07	2073,20 ± 844,80	Time	0,01	0,91	
	Control	1832,45 ± 961,50	1908,79 ± 357,46	Time*group	0,67	0,41	
	Total	2012,24 ± 933,15	2007,83 ± 694,90	Group		0,06	

Time^*^group: interactional, mean (+SD) F: Mix Repeated MeasuresAnova (Sphericity Assumed).

The findings indicate that a significant group-by-time interaction effect was found for Energy Intake (EI) (*F* (1,81) = 20.18; *p* < 0.05, Partial Eta Squared = 0.20; large). Following Bonferroni correction for pairwise comparisons, it was observed that EA in the experimental group increased from pre-intervention 1739.7 ± 396.6 kcal to post-intervention 2046.1 ± 448 kcal (*p* = 0.01).

A significant group-by-time interaction effect was found for protein intake (*F* (1,81) = 6.71, *p* < 0.05, Partial Eta Squared = 0.08; medium). Following Bonferroni correction for pairwise comparisons, it was observed that protein intake in the experimental group increased from pre-intervention (70.66 ± 22.06) to post-intervention (84.76 ± 22.53) (*p* = 0.01). Similarly, a significant group-by-time interaction effect was found for carbohydrate intake (*F* (1,81) = 6.48, *p* < 0.05, Partial Eta Squared = 0.07; medium). Following Bonferroni correction for pairwise comparisons, it was observed that carbohydrate intake in the experimental group increased from pre-intervention (184.36 ± 54.65) g to post-intervention (228.04 ± 57.09) g (p = 0.01).

A significant group-by-time interaction effect was found for fat intake (*F* (1,81) = 6.17, *p* < 0.05, Partial Eta Squared = 0.07; medium). Following Bonferroni correction for pairwise comparisons, it was observed that fat intake in the experimental group increased from pre-intervention (76.52 ± 33.64) g to post-intervention (82.80 ± 25.91) g (*p* = 0.01).

There was no group-by-time interaction for fiber, calcium, iron, and water intake (Table 3).

Furthermore, at the beginning of the season, both groups nearly met their iron requirements according to the Recommended Daily Allowance – (RDA) for adolescent girls, which is 15 mg. However, they were unable to adequately meet their iron needs during the season (29). Calcium intake for both control and intervention groups (1009.3 mg ± 392.1 mg, 1,096 mg ± 431.7) was lower than RDA for adolescent female athletes (1,300 mg) (34).

The prevalence of athletes with Low Energy Availability (LEA), according to energy availability calculations in the whole group, was 73.3% for the intervention group (n = 33) and 52.6% for the control group (*n* = 20) at the beginning of the study (Table 4). Six months after the intervention, this was measured as 46.7% (*n* = 21) in the intervention group, while it remained at 52.6% (*n* = 22) in the control group.

**Table 4:** Proportion of athletes with low, decreased, and normal energy availability and proportions in LEAF-Q and EAT-26 scores of participants pre-post intervention divided by intervention and control group.

Pre/Post-intervention	Parameter	Group				Total	
		Intervention		Control			
		*N*	%	*N*	%	*N*	%
Pre-intervention	LEAF-Q (<8)	22	48,8	14	36,8	36	43,3
	LEAF-Q (≥8)	23	51,2	24	63,2	47	56,6
Post-intervention	LEAF-Q(<8)	25	55,5	13	34,2	38	45,7
	LEAF-Q(≥8)	20	45,5	25	65,8	45	54,3
Pre-intervention	EAT-26 (<20)	26	57,7	20	52,6	46	55,4
	EAT-26 (≥20)	19	42,3	18	47,4	37	44,5
Post-intervention	EAT-26 (<20)	27	60	23	60,5	50	60,2
	EAT-26 (≥20)	18	40	15	39,5	33	39,8
Pre-intervention	EA ≤ 30 kcal/kg/FFM EA = 31–45 kcal/kg/FFM EA > 45 kcal/kg/FFM	33 10 2	73,3 22,2 4,4	20 14 4	52,6 36,8 10,5	53 24 6	63,8 28,9 7,3
Post-intervention	EA ≤ 30 kcal/kg/FFM EA = 31–45 kcal/kg/FFM EA > 45 kcal/kg/FFM	21 18 6	46,7 40 13,3	22 13 3	57,8 34,2 7,9	43 31 9	51,8 37,3 10,0

The prevalence of athletes with disordered eating attitudes (EAT-26 > 20) was 42.3% (*n* = 19) in the intervention group and 47.4% (*n* = 18) in the control group during the pre-intervention. It decreased to 40% (*n* = 18) in the intervention group and was measured as 39.5% (*n* = 15) in the control group during the post-intervention (Table 4).

The prevalence of athletes with Low Energy Availability (LEA), determined by LEAF-Q scores, was 51.2% (*n* = 23) for the intervention group and 63.2% (*n* = 24) for the control group during the pre-intervention. In the post-intervention. it was measured as 45.5% (*n* = 20) for the intervention group and 65.8% (*n* = 25) for the control group.

Table 5 shows the changes in LEAF-Q, EAT-26, and SNKQ scores of the athletes before and after the intervention in both groups.

**Table 5:** Changes in LEAF-Q, EAT-26, and SNKQ scores of the athletes before and after the intervention in both groups.

Questionnaire	Group	Pre	Post		*F*	Sig.	Partial Eta Squared
LEAF-Q	Interv.	8,57 ± 4,36	6,82 ± 3,72	Time	1,97	0,16	
	Control	10,21 ± 7,41	13,91 ± 9,07	Time^*^group	15,50	0,00	0,16
	Total	9,23 ± 5,80	9,67 ± 7,28	Group	0,30	0,001	0,12
EAT-26	Interv.	17,86 ± 11,01	17,98 ± 10,59	Time	0,10	0,75	
	Control	18,41 ± 10,02	17,72 ± 10,24	Time^*^group	0,21	0,65	
	Total	18,07 ± 10,57	17,88 ± 10,39	Group		0,94	
SNKQ	Interv.	29,18 ± 8,60	35,29 ± 7,17	Time	9,19	0,00	0,10
	Control	27,21 ± 9,77	27,03 ± 9,74	Time^*^group	10,35	0,00	0,11
	Total	28,39 ± 9,08	31,96 ± 9,20	Group	3,20	0,03	0,09

Time^*^group: interactional, mean (+SD) F: Mix Repeated MeasuresAnova (Sphericity Assumed).

The LEAF-Q scores indicate a significant group-by-time interaction effect (*F*(1,81) = 15.50; *p* < 0.05, Partial Eta Squared = 0.16). Following Bonferroni correction in pairwise comparisons, it was observed that LEAF-Q scores in the experimental group decreased from pre-intervention (8.57 ± 4.36) to post-intervention (6.82 ± 3.72) (*p* = 0.01).

Similarly, SNKQ scores show a significant group-by-time interaction effect (*F* (1,81) = 10.35, *p* < 0.05, Partial Eta Squared = 0.11). Following Bonferroni correction in pairwise comparisons, it was observed that SNKQ scores in the experimental group increased from pre-intervention (29.18 ± 8.60) to post-intervention (35.29 ± 7.17) (*p* = 0.01).

There was no group-by-time interaction for EAT-26 scores (*F* (1,81) = 0.21, *p* = 0.65).

## 4 Discussion

The primary outcome of this study indicates that nutrition education significantly enhanced nutrition knowledge among young female athletes. This improvement in knowledge may have affected various factors, including energy availability, energy intake, protein, carbohydrate and fat intake, body mass index, and fat mass in young female elite athletes. Notably, the intervention resulted in a 7.2% improvement in the Sports Nutrition Knowledge (SNK) of the participants. However, it’s noteworthy that the number of correct answers in the SNK questionnaire for both the control and intervention groups remained below 50% (mean score and percentage for control and intervention groups, respectively, after the intervention were 27.0 (32.5%) and 35.3 (34.9%), with a total score of 78), emphasizing the need for further improvement. In a study by Condo et al., the median score for total sports nutrition knowledge among Australian female football players was found to be 54,5% (30).

Initially, the study identified a high prevalence of Low Energy Availability (LEA) among athletes, measured at 63.8%. The use of the LEAF-Q questionnaire, an indirect method, showed a similar result of 56.6%. This highlights the practicality of the LEAF-Q in identifying athletes with LEA symptoms, as direct methods measuring energy intake and expenditure are challenging and often inaccurate. Some studies show that self-reporting resulted in an underestimation of EI by 5–21% (31), while the use of metabolic equivalents (METs) overestimated resting metabolic rate by 20% (32). Cross-sectional studies indicate that the estimation of low EA in athletes varies from 51 to 63% (25, 33). In a study conducted by Łuszczki et al. (34) it was found that 64.7% of participants were classified as being at risk for low energy availability according to their LEAF-Q scores among young football players. The researchers noted that the mean age of the study group was lower (15,41 years) than most in the literature (35) which could be a probable explanation for the lower level of sports nutrition knowledge among this athlete group.

In our study, we found that the median energy intake among all athletes was 1863.4 ± 463.2 kcal, significantly lower in the intervention group at 1739.7 ± 396.6 kcal initially. After nutrition education, there was a notable improvement, with an increase of 307 kcal/day and a positive impact on energy availability (+8.1 kcal/kg FFM/day). Łagowska et al. (36) reported improved energy intake (+234 kcal/day) and energy availability (+7.5 kcal/kg FFM/day) among 45 female athletes with menstrual dysfunction after a three-month intervention. In this intervention, athletes were informed of nutritional mistakes and provided with individualized diets (40). After extending the study to 9 months, the researchers reported a more profound increase in energy intake and energy availability (37).

In our study, carbohydrate intakes, respectively, among the control and the intervention group were 212 g (3,57 g/kg/day) and 184.4 g (3,14 g/kg/day) in the beginning and improved significantly in the intervention group 221.6 g (3,76 g/kg/day) *p* = 0.125, 227.7 g (3,82 g/kg/day) *p* = 0.039. The daily mean carbohydrate intake in female Australian football players has been found as 3 g/kg/day therefore below the minimum carbohydrate recommendation for a moderate exercise intensity of approximately 1 h per day (5–7 g/kg/day) (30).

At baseline, the daily mean protein intake for the control and the intervention groups were 1,29 g/kg/day and 1,20 g/kg/day, respectively. After the intervention, protein intake increased to 1,30 g/kg/day and 1,42 g/kg/day (30). Protein intake before and after intervention, across both study groups, was consistent with current recommendations (1.2–2.0 g/kg/day) (3).

Sports nutrition education interventions are recommended to help athletes understand their advanced dietary requirements and provide practical strategies to meet these dietary recommendations. Our study employed a nutrition education intervention consisting of six physical sessions totaling 360 min, a strategy proven effective in similar studies with contact time ranging from 90 min (38) to 390–490 min (39) and the total number of sessions ranging from one (40) to ten (39). This approach resulted in a 7.2% improvement in SNK, which is consistent (8.3%) with a similar study conducted with adolescent swimmers (41). Previous studies investigating the effectiveness of nutrition education approaches have reported similar improvements in athlete nutrition scores, ranging from 6.4–25.2% (42–44).

Another remarkable finding in the current study was the high prevalence of disordered eating attitudes among female adolescent athletes (44,5%). This finding is close to the prevalence rate found in a study performed in Jordan which reported a rate of 34% (45). In a study performed by Raymond-Barker et al. (46), it was found that the lack of nutrition knowledge was not associated with increased disordered eating behavior, as measured by the EAT-26 questionnaire. This suggests that not only nutrition knowledge but other factors may play a role in the development of disordered eating attitudes and eating disorders. Wells et al. (47) claimed that one-third of the athletes surveyed reported being told to alter their body weight for their sport in order to improve performance. This demonstrates that the pressure on the athletes regarding their body shape or composition may be a strong contributor to the risk of DE in order to satisfy their coach and improve their athletic abilities. External pressure for athletic success is also thought to contribute to an increased risk of DE. Parental pressure, peer influence, and the media have been associated with DE, along with body dissatisfaction (48), and specifically, peer influence has been found to be a significant predictor of bulimic behaviors (49).

According to various studies, young athletes who report disordered eating attitudes consume less than the required energy through their diet (50, 51). Athletes who choose unhealthy ways to control their weight have been found to have lower energy availability than those who do not engage in unhealthy behaviors (52). Using the commonly agreed-upon limit of 30 kcal/kg FFM/day, studies have found that 30 to 60% of athletes experience low energy availability (52–55). It seems that a significant number of athletes do not consume enough energy, necessary not only to cover their training demands but most importantly for the maintenance of their normal physiological functions. This situation may cause medical complications in several body systems and impair growth and maturation (56–58).

This is the first randomized controlled nutrition education intervention study on energy availability, sports nutrition knowledge, and dietary attitude in young female endurance athletes furthermore it included a control group in contrast to other intervention studies involving female athletes. Second, it is conducted with young female athletes for whom the literature lacks sufficient information. Third, due to the face-to-face lectures in sports clubs within a group, given by a dietitian, athletes had the chance to take notes, ask questions, and discuss in a group setting with their coaches’ participation. This could further positively affect nutrition behavior, as coaches are one of the primary sources of nutrition knowledge for young athletes (59).

Another strength of the study was, after the dropout (*n* = 17) at the beginning of the study, there were no athletes who did not attend the nutrition education sessions. This means that 100% of all athletes took part in the nutrition education sessions, despite the physical nature of these sessions, which may have increased the effectiveness of the intervention.

A limitation of the current study is the use of self-reported dietary intake, which may be subject to underreporting by participants (60). Therefore, there is a potential bias for an overestimation in the prevalence of LEA. Furthermore, the 3-day monitoring period may not adequately reflect the normal dietary habits and activity levels of the athletes throughout the entire season. Additionally, energy expenditure was calculated based on the registered estimated activity levels, not using indirect calorimetry or acceloremeters due to the associated expenses and impracticality to use in every athlete, especially given the high sample size (*n* = 83). Furthermore, it would be useful to apply these questionnaires to assess the knowledge level of trainers who may have a significant impact on the athletes in terms of sports nutrition knowledge, nutrition behaviour, eating attitude and body composition.

## 5 Conclusion

The nutrition knowledge level of female adolescent athletes was found to be low in this study, and with the nutrition education intervention, it has been significantly increased. The outcomes of the present investigation indicate that the young female athletes in this study exhibit inadequate intake of carbohydrates, iron, and calcium in relation to the current guidelines for athletes. We recommend the inclusion of individually tailored education programs given by a sports dietitian, to highlight the importance of dietary intake on body composition and performance. These programs should also provide practical strategies to achieve recommended dietary intake for athletes, their families and other team members engaging with the athletes. This aims to increase awareness about LEA and take measures before the progression of risk factors. Furthermore, avoiding negative comments and pressure on body weight in the context of sports and the family environment, along with providing information on professionals who can advise athletes on nutrition and eating disorders is also important.

## Data availability statement

The datasets presented in this article are not readily available due to restrictions (e.g., their containing information that could compromise the privacy of research participants). Requests to access the datasets should be directed to CTA, diyetisyencansutek@gmail.com.

## Ethics statement

The study is been registered to Clinical Registry with the number (NCT06116097). The study involving humans were approved by Research Ethics Committee of Istanbul Medipol University with the number (E.8234/196) in 2019. The study was conducted in accordance with the Declaration of Helsinki. Written informed consent for participation in this study was provided by the participants’ legal guardians/next of kin and they were allowed to drop out of the study at any time.

## Author contributions

CT: Data curation, Formal analysis, Investigation, Methodology, Writing – original draft. CG: Data curation, Investigation, Methodology, Writing – original draft. GE: Investigation, Methodology, Supervision, Writing – review & editing.
